# Why Are Some Children More Sick Than Others? Lessons From Genome-wide Association Studies of Respiratory Viral Infections

**DOI:** 10.1097/INF.0000000000005104

**Published:** 2025-12-10

**Authors:** Pekka Vartiainen, Santtu Heinonen

**Affiliations:** From the * Institute for Molecular Medicine Finland (FIMM), HiLIFE, University of Helsinki, Helsinki, Finland; † Centre for Fertility and Health, Norwegian Institute of Public Health, Oslo, Norway; ‡ Finnish Vaccine Research (FVR), Tampere; § Pediatric Research Center, Helsinki University Hospital, Faculty of Medicine, University of Helsinki, Helsinki, Finland.

**Keywords:** genetics, respiratory infections, viruses, genome-wide association study

Viral respiratory infections certainly impact every child, but it might seem that some children are more often and more severely ill than others. Outside of clinically recognized and mechanistically explained immunodeficiencies, either inborn or acquired, the variation in infection susceptibility remains rather poorly understood. Clinical experience might suggest that, for example, the older sibling’s infection history can be predictive for the younger sibling as well. What has the dynamic field of human genetics discovered related to this varying infection susceptibility?

In this review, we describe germline genetic variation associated with susceptibility to viral respiratory infections in childhood. We focus on those findings from population-based genome-wide association studies (GWAS), where the GWAS association with infectious phenotype has been explained, or has helped to explain, the gene-phenotype associations at a mechanistic level. The goal of this review is, rather than listing single nucleotide polymorphisms for clinicians to memorize, to familiarize the reader of the strengths and limitations of GWAS through specific childhood infection examples.

To ensure focus, we exclude chromosomal or multisystem syndromes (eg, trisomy 21) and major monogenic disorders (eg, cystic fibrosis), in which recurrent infections are a feature. We also exclude inborn errors of immunity and rare variants with high impact and penetrance, which are typically described as case and family studies of very severe infections or as monogenic causes of severe diseases. Here, the interested reader is referred to, for example.^1–3^ We start with pathogen-specific associations, mention more general susceptibility factors and close with a brief discussion on the next steps of clinically relevant research directions.

## PATHOGEN-SPECIFIC ASSOCIATIONS

### Rhinoviruses can be Mediators of Genetic Risk of Asthma

Host genetic predisposition to virus infections, most notably to rhinovirus (RV), has revealed interesting mechanistic explanations on why and how some genes are associated with asthma.

The *17q12-21* locus (region in the chromosome 17, later referred to as 17q) contains the first discovered and one of the most replicated genetic associations with childhood asthma.^4^ 17q contains many genes (eg, *ORMDL3, GSDMB* and *IKZF3*), and variants in these genes show strong linkage disequilibrium (LD), meaning that the variants are often inherited together and strongly associated with each other at the population level. This makes identifying the true causal variants very difficult. 17q variants are also risk factors for early RV wheezing illnesses.^5^ Furthermore, it appears that the effect of 17q variants on asthma is modified by virus infections and environmental exposures,^4,5^ asthma risk associated with the 17q variants being stronger in children with early RV wheezing. This can be a sign of gene-environment interaction, or a result from varying penetrance (ie, a polymorphism not always leading to observed phenotype changes), or that there are other factors explaining both RV and asthma susceptibility. Importantly, the effect of 17q locus variants appears to be much smaller in persons with African ancestry, at least partly due to different LD structure of the 17q region,^6^ but it is also speculated that different environmental exposures, or different manifestation of gene-environment interactions,^4,7^ might play a role. Such differences in genetic effects according to ancestry are in fact, very important in genetic research, and will be discussed later.

*CDHR3* variation (rs6967330) was originally discovered to be associated with severe childhood asthma exacerbations.^8^ Later works showed CDHR3 protein, expressed in epithelial cell surfaces, to be a rhinovirus C (RV-C) receptor, and that asthma-associated variants caused overexpression of CDHR3 in airway epithelial cells, in turn leading to increased RV-C binding and replication.^9^ It is thought that this increased RV-C predisposition is the mechanistic explanation why *CDRH3* overexpression is associated with asthma.

Very interestingly, in vitro infection of airway epithelial cells with rhinovirus was shown to cause up-regulation in expression of over 40 asthma-associated genes in nonciliated airway cells.^10^ This suggests that RV could be a triggering factor for asthma, instead of only reflecting underlying predisposition; however, causation has not been established. Research of this kind is useful because most GWAS signals are in noncoding regions and do not point directly to a single nearby gene; their effects often act through complex regulatory elements and modest shifts in gene expression networks.

The previous examples provide concrete cases where linking the host genotype to infection phenotypes has helped clarify the pathways underlying single nucleotide polymorphism-level associations. They offer support, but do not confirm, causality of respiratory viruses and asthma, and factors such shared susceptibility and ascertainment remain plausible.

### Still no Discoveries About Genetic Predisposition to RSV Infections

Respiratory syncytial virus (RSV) is the most common cause of severe respiratory infections in early childhood. Like rhinovirus, RSV infections are similarly associated with later asthma, but no convincing genetic associations with RSV infections have been identified,^11–14^ and RSV is thought to be more causally attributed to asthma.^15^ Previously described 17q12-21 locus and CDHR3 variants seem to be associated only with non-RSV bronchiolitis (typically caused by rhinovirus), and not with RSV bronchiolitis.^12^ Several suggestive genetic associations with RSV have been reported in both GWASs^11–14^ and other candidate gene studies, but the lack of replication warrants caution in their interpretation, as such associations may represent statistical noise that fails to replicate in better-powered studies.

The most conservative reading of current RSV GWAS’s is not that host genetic effects are negligible, but that available studies are underpowered and phenotypically noisy for the effect sizes we should expect. If true, common-variant effects on RSV severity lie in the odds ratio ≈1.05–1.15 range, genome-wide discovery typically requires tens of thousands of well-phenotyped cases, thresholds that few pediatric cohorts currently meet. In addition, registry-based RSV endpoints are a mix of different exposure, testing and admission practices, which further masks possible signals. Because severe RSV risk depends strongly on age and prematurity, RSV severity could, hypothetically, serve as a window into early immune system maturation or response characteristics, enabling genetic analyses of immune phenotypes that are otherwise hard to measure. Observed differences in natural killer cell responses according to RSV severity^16^ further support such hypotheses.

### Lessons From COVID-19

COVID-19 is usually mild in children, but in adult populations, very large international consortia have worked successfully to find genetic liability for COVID-19. One of them, the COVID-19 Host Genetic Initiative, identified 51 distinct genome-wide significant loci.^17^ One of the contributions of the study was a systematic examination of infection susceptibility (the probability of acquiring an infection) and severity (the clinical course of a disease once infected). In COVID-19, susceptibility loci were often mapped to viral entry and airway defense pathways, whereas disease severity loci were primarily mapped to the type I interferon pathway.

Specific for children, COVID-19 can infrequently lead to severe Multisystem Inflammatory Syndrome in Children. More detailed sequencing in combination with advanced computational methods has revealed rare variants, for example, in *BTNL8* gene functioning in the gut lining immunity, that associate with Multisystem Inflammatory Syndrome in Children.^18^

## BEYOND PATHOGEN-SPECIFIC ASSOCIATIONS

### The HLA Region

The human leukocyte antigen (HLA, or Major Histocompatibility Complex) region consists of multiple genes in chromosome 6 that code molecules responsible of binding and presenting antigens as part of the immune system. The HLA region is extremely polymorphic (the genes have a lot of variation), this diversity being 1 aspect of human immunity, and the HLA variants display high LD (similarly to 17q). These aspects make studying the HLA region with GWAS methods very challenging.^19^ Nevertheless, variation in HLA is associated with many immune-related phenotypes, including also respiratory infections.^20,21^ An article comparing multiple infection phenotypes reported that susceptibility to certain viral infections (eg, mumps, chickenpox and mononucleosis) was more linked to variation in HLA class I, and bacterial phenotypes were more connected to HLA class II.^20^ However, the specific mechanisms and significance of these associations, and whether the HLA region exerts any childhood-specific effects, remain unknown. This HLA complexity also calls for more comprehensive phenotype definitions.

### The Complex Chain of Secretor Status, Blood Groups and Gut Microbiome

Histo-blood group antigens (HBGAs) are carbohydrate structures on red blood cells (forming the basis of ABO blood groups), mucosal epithelia, and secretions. Genetic variation in the enzymes constructing HBGAs can change HBGA expression, which in turn results in varying infection predisposition. FUT2 enzyme determines secretor status: loss-of-function (“non-secretor”) abolishes mucosal H antigens and gives strong protection against norovirus^22^ and some strains of rotavirus^23^; conversely, secretors show higher risks for otitis media,^20^ respiratory tract infections,^24^ childhood asthma and *Streptococcus pneumoniae* infections.^25^ A very interesting analysis showed that genetic variation in the ABO blood group, in interaction with FUT2 secretorship status, is highly associated with a specific gut microbiota signature. This variation affects the host’s (ie, human’s) carbohydrate secretion, which in turn enables certain microbes that utilize these carbohydrates to grow and thrive.^26^ This exemplifies a specific pathway through microbes that connects host genetic variation to not only infections but also more broad health endpoints associated with the ABO blood group.

### Ancestry

Over time, different populations have undergone bottlenecks and expansions, migrated and admixed, and evolved distinct recombination patterns. Such forces shape allele frequencies and haplotype lengths, so the variants that travel together (ie, LD) can look different across ancestries. Most GWAS studies and genotyped cohorts over-represent persons with European descent, and many discovered genetic associations have not been replicated in other, non-European ancestries. For example, the 17Q12-21 loci effects on asthma are weak in African populations, and in Asian populations, variation in IFITM3 gene (*rs12252-C*) clearly modulates influenza severity, but the allele is much rarer and the effect more unclear in European cohorts.^27^ Ethnically diverse genotyped cohorts and ancestry-specific genotype reference panels are very important in ensuring representative genetic research.^28^ Furthermore, as several infectious disease phenotypes are associated with ethnic background, or the diseases and epidemics can be geographically limited to certain populations, and – most importantly – the burden of many infectious diseases is the greatest in non-European populations, proper examination of population- and ancestry-specific effects will maximize our chances of discovery in future genetic research of infection predisposition.^2,29^

### Next Steps

In this review, we have described some common genetic variants that have been discovered in a GWAS setting to be associated with respiratory infection phenotypes. Some of these discovered associations have also helped to pinpoint causal pathways and mechanisms behind gene-phenotype associations. Such discoveries, but also the limitations observed in these and other studies, are important lessons concerning genetic studies.

Genetic liability typically arises from thousands of variants with small effects. Rather than single-variant tests, genome-wide aggregation methods such as polygenic risk scores can be informative at the population level. It remains to be seen whether polygenic risk scores, maybe in combination with large-effect rare variants, could be used in targeting prevention efforts – given proper validation in all ancestries.^2,29^ In contrast to large population level analyses, improved multiomic approaches, involving gene regulation pathways, and gene, RNA and protein expression at tissue or even single-cell level, can help in understanding mechanisms behind gene-phenotype associations in close detail. The immune system is an extremely complex network of multiple brakes and accelerators, highlighted by for example, the complexity of the HLA region, and studying it will require significant advances in both data generation and analysis. Although not covered here, monogenic inborn errors of immunity can serve as further clues in understanding the regulation of immune mechanisms.

### Phenotype Definition and Child Representation in Biobank Research Are Targets of Improvement

Biobank data is rapidly growing in sample sizes, data richness and diversity. Even the most advanced multiomics analyses that integrate networks of intermediate phenotypes (such as molecular, imaging and physiological traits) remain dependent on accurate phenotype and disease definitions. Better phenotype definitions are a practical step to increase the chances of discovery in host genetic infection research. Respiratory infections, or arguably any infections, are not well captured in most routinely collected datasets, such as diagnoses and registries. Improved phenotype definition could mean, for example, quantification of the host response (“immunomics”), or better capturing of microbes, in particular the microbe genotypes and the interaction with host and microbe genomics (see eg,^14^ related to RSV).

Lastly, children are underrepresented in biobank research. Major population-based biobanks are adult cohorts, and pediatric biobank participation remains limited due to consent and operational barriers. More early sample collections, adequate representation, longitudinal follow-up, and explicit prioritization on child-relevant phenotypes are ways to ensure that also children will benefit from advancing -omics research. This matters across most pediatric conditions, but as childhood is the peak window for infections, such efforts are likely to unlock infection-specific genetic insights with potential implications for prevention and treatment.
